# Ischemic stroke is associated with the pro-inflammatory potential of *N*-glycosylated immunoglobulin G

**DOI:** 10.1186/s12974-018-1161-1

**Published:** 2018-04-26

**Authors:** Di Liu, Zhongyao Zhao, Anxin Wang, Siqi Ge, Hao Wang, Xiaoyu Zhang, Qi Sun, Weijie Cao, Ming Sun, Lijuan Wu, Manshu Song, Yong Zhou, Wei Wang, Youxin Wang

**Affiliations:** 1 Beijing Key Laboratory of Clinical Epidemiology, School of Public Health, Capital Medical University, 10 Youanmen, Beijing, 100069 China; 2 Department of Neurology, Beijing Tiantan Hospital, Capital Medical University, Beijing, China; 3 School of Medical and Health Sciences, Edith Cowan University, Perth, WA 6027 Australia; 4 Beijing Institute of Heart, Lung and Blood Vessel Diseases, Beijing Anzhen Hospital, Capital Medical University, Beijing, 100029 China

**Keywords:** Ischemic stroke, Cerebral arterial stenosis, Immunoglobulin G, *N*-glycans, Inflammation markers

## Abstract

**Background:**

Glycosylation significantly affects protein structure and function and thus participates in multiple physiologic and pathologic processes. Studies demonstrated that immunoglobulin G (IgG) *N*-glycosylation associates with the risk factors of ischemic stroke (IS), such as aging, obesity, dyslipidemia, type 2 diabetes, and hypertension.

**Methods:**

The study aimed to investigate the association between IgG *N*-glycosylation and IS in a Chinese population. IgG glycome composition in patients with IS (*n* = 78) and cerebral arterial stenosis (CAS) (*n* = 75) and controls (*n* = 77) were analyzed by ultra-performance liquid chromatography.

**Results:**

Eleven initial glycans and 10 derived glycans in IgG glycome representing galactosylation, sialylation, and bisecting GlcNAc significantly differed between IS patients and CAS and healthy controls after controlling for gender, age, obesity, diabetes, hypertension, and dyslipidemia. Logistic regression models incorporating IgG glycan traits were able to distinguish IS from CAS (area under receiver–operator characteristic curves (AUC), 0.802; 95% confidence interval (CI), 0.732–0.872) and controls (AUC, 0.740; 95% CI, 0.661–0.819). The canonical correlation analysis indicated that initial *N*-glycan structures are significantly correlated with inflammation markers (*r* = 0.566, *p* < 0.001).

**Conclusion:**

Our findings indicated that loss of galactose and sialic acid, as well as addition of bisecting GlcNAc, might involve in pro- or anti-inflammatory IgG functionality and further contribute to the pathogenesis of IS. IgG glycan profiles may be developed as clinical useful biomarkers for chronic disease in the future.

**Electronic supplementary material:**

The online version of this article (10.1186/s12974-018-1161-1) contains supplementary material, which is available to authorized users.

## Background

Stroke is one of the leading causes of death and permanent disability worldwide [1, 2]. Approximately 80% of stroke is ischemic, whereas 20% is due to primary hemorrhage [3]. Ischemic stroke (IS) is a multifactorial and complicated disease, resulting from an intricate interplay between environmental and genetic factors [3]. Cerebral arterial stenosis (CAS) of the major arteries in the brain is one of the most common pathomechanisms for the development of ischemic stroke [4]. There is a pressing need for better understanding and prevention of CAS. CAS and IS are characterized by a pro-inflammatory state and elevated levels of markers of inflammation, such as C-reactive protein and interleukin-6, which are associated with the risk of CAS and IS [5–8]. Although increasing evidence has indicated that inflammation is important in the progression of CAS to IS [7, 8], and numerous risk factors have been identified, the underlying mechanisms of IS remain largely unknown.

By attaching to individual proteins, glycans participate in many key biological processes, including cell adhesion, molecular trafficking and clearance, receptor activation, signal transduction, and endocytosis [9, 10]. Glycans were also found to be involved in the pathophysiology of some major diseases, such as inflammatory diseases and cancers [11–13]. Glycans have a particularly important role in the immune system, and glycosylation may affect the function of the immune system on multiple levels [13, 14]. As glycans do not have a direct genetic template, glycan structures are determined by complex dynamic interactions between a number of genetic and environmental factors [15, 16]. These unique qualities have promoted interest in the role and use of *N*-glycans as potential predictors of the development of complex diseases such as IS.

Immunoglobulin G (IgG) plays an important role in the human immune system, and *N*-glycans attach to the conserved asparagine 297 in the fragment crystallizable (Fc) region and act as a switch between pro- and anti-inflammatory IgG functionality [17]. Changes in IgG glycosylation are not only involved in several inflammatory diseases [18–21] but also can be a part of the molecular mechanism leading to the promotion of inflammation [22–24]. The inflammatory role of IgG *N*-glycosylation, together with its association with the risk factors of ischemic stroke, such as aging, obesity, dyslipidemia, type 2 diabetes (T2D), and hypertension [25–28], led us to hypothesize that the changes in IgG *N*-glycan profiles are involved in the pathogenesis of IS by regulating the inflammatory response.

In this study, we investigated the association of IgG *N*-glycan profiles with CAS and IS to provide biomarkers for the screening of CAS and IS. In addition, we aimed to determine the association between IgG *N*-glycome and markers of inflammation, including C-reactive protein (CRP), tumor necrosis factor-alpha (TNF-α), and matrix metalloproteinase-9 (MMP9), to further elucidate the roles of IgG glycosylation on CAS and IS.

## Methods

### Participants

All participants were recruited from the *Jidong* Oil-field Hospital, Chinese National Petroleum, and the *Tiantan* Hospital, Capital Medical University from September 2009 to September 2013 [29, 30]. All participants were required to meet the following inclusion criteria: (1) signed informed consent prior to participation; (2) 18 years or older aged equal to or greater than 18 years old. Individuals were excluded based on the following criteria: (1) pregnant or lactating women; (2) history of mental illness or infectious disease; (3) history of aneurysm caused by cerebral infarction, cerebral hemorrhage or other cerebrovascular diseases, congenital heart disease, acute myocardial infarction, liver disease, renal failure, malignant tumor, chronic obstructive pulmonary disease, rheumatoid arthritis, or other diseases; and (4) history of medications during the preceding 2 weeks in the controls. Written informed consent was obtained from each subject at the beginning of the study.

### Diagnosis of IS and CAS

IS patients, diagnosed according to the International Classification of Disease (9th revision, codes 430 to 438) on the basis of history, clinical symptoms, physical examination, and cranial computed tomography (CT) or magnetic resonance imaging (MRI) [31], were included in this study. CAS was diagnosed based on the level of the vessel stenosis according to the established criteria [30, 32]. The age- and sex-matched controls were defined as no history of CAS and stroke and no family history of stroke.

### Covariates

Demographic characteristics of participants, including age, gender, and ethnicity, were collected by a questionnaire. Detailed information about demographic characteristics has been described previously [29, 30]. Weight and height measurements were carried out with the participants wearing only light indoor clothing and without shoes. The body mass index (BMI) was calculated by the formula weight (in kilograms)/height^2^ (in meters squared). Obesity is defined as BMI > 28.0 kg/m^2^ [33]. Systolic blood pressure (SBP) and diastolic blood pressure (DBP) were measured twice on the right arm using a standard mercury sphygmomanometer on set with the subjects resting at least 10 min in a sitting position (World Health Organization (WHO), [34]). The participants were then classified into the hypertension group (mean SBP ≥ 140 mmHg or mean DBP ≥ 90 mmHg) or the normal blood pressure group (mean SBP < 120 mmHg and mean DBP < 80 mmHg) [34].

After an overnight fasting, two tubes of blood (5 mL) were collected in the morning by venipuncture. One sample was taken in vacuum negative pressure tubes not containing ethylene diamine tetraacetic acid (EDTA) to acquire serum (2 mL), which was used to measure the blood biochemistry indexes and inflammatory mediators, and the other sample was taken in vacuum negative pressure tubes containing EDTA. Then, the whole blood was centrifuged at 3000 rpm for 10 min, and the plasma (3 mL) was separated which was used to detect *N*-glycomic. All collected blood samples were processed within 8 h and stored at − 80 °C until further measurement.

The fasting blood glucose (FBG) concentrations were measured by the glucose oxidase-peroxidase method (Mind Bioengineering Co. Ltd., Shanghai, China). Diagnosis of T2D was made by physicians according to the 1999 WHO Criteria (FBG greater than or equal to 7.0 mmol/L) [35]. Serum total cholesterol (TC), triglycerides (TG), high-density lipoprotein (HDL) cholesterol, and low-density lipoprotein (LDL) cholesterol were measured using previously described methods with an Olympus Automatic Biochemical Analyzer (Hitachi 747; Hitachi, Tokyo, Japan). According to the guideline for the prevention and control of dyslipidemia of adults in China [36], the participants were grouped into dyslipidemia with TC ≥ 6.2 mmol/L, or TG ≥ 2.3 mmol/L, or HDL < 1.0 mmol/L, or LDL ≥ 4.1 mmol/L.

### Measurement of inflammatory mediators

The level of inflammatory mediators in the serum was measured using the relevant enzyme-linked immunosorbent assay (ELISA) kit for CRP, TNF-α, and MMP9. The inflammatory mediators were detected in accordance with the protocols from the CRP kit (R&D Systems, Bio-Techne China Co. Ltd., Beijing, China), TNF-α kit (R&D Systems, Bio-Techne China Co. Ltd., Beijing, China), and MMP9 kit (R&D Systems, Bio-Techne China Co. Ltd., Beijing, China) as previously reported [37].

### IgG *N*-glycans analysis

As previously reported, IgG was isolated from human plasma firstly [38]. In brief, after washing and equilibrating protein G monolithic plates, 50 μL of plasma was diluted 10× with binding buffer (1× phosphate-buffered saline, pH 7.4), applied to the protein G plates, and washed instantly. IgGs were eluted with 1 mL of 0.1 M formic acid and immediately neutralized with 1 M ammonium bicarbonate. Then, IgG *N*-glycans were released and labeling was performed. The released *N*-glycans were labeled with 2-aminobenzamide, a fluorescent dye used to make glycans visible by ultra-performance liquid chromatography (UPLC), by multistage mixing with 2-aminobenzamide, dimethylsulfoxide, glacial acetic acid, and 2-picoline borane. In the end, IgG *N*-glycans were analyzed by hydrophilic interaction chromatography (HILIC)-UPLC into 24 IgG glycan peaks (GPs) [38]. The glycan structures of the most abundant glycans per peak were reported previously [25].

All chromatograms were separated in the same manner into 24 peaks, and the amount of glycans in each peak was expressed as a percentage of total integrated area. An additional 54 derived traits describing the relative abundances of galactosylation, sialylation, bisecting GlcNAc, and core fucosylation were calculated by the remaining 24 directly measured glycans. Of the derived traits, 40 were of interest and included in the analysis. Normalization and batch correction of UPLC data were detailed in a published study [38].

### Statistical analysis

Normality distributions of inflammation markers and glycans were tested by the Kolmogorov–Smirnov tests, of which *p* < 0.10 was considered statistically significant. Continuous variables underlying normal distribution were represented as the mean ± standard deviation (SD); otherwise, medians (*P*_25−_ *P*_75_) were used. The differences of continuous variables among three groups were tested by one-way analysis of variance (ANOVA) or the Kruskal–Wallis test. Categorical variables were represented as *n* (proportion), and the differences among the three groups were tested by chi-square tests.

For markers of inflammation and most of the glycans that were not normally distributed, normalized transformations were applied. The Kruskal–Wallis test was performed to compare the differences of initial and derived IgG glycans among the three groups. Multinomial logistic regression was performed to identify 24 initial glycans and 54 derived glycans related to disease status, adjusting the effect of age, sex, obesity, diabetes, hypertension, and dyslipidemia. The significant glycans were graphed by forest plot, which was generated using the *R* package “forestplot.” Then, a logistic regression model assessed the discrimination of IS, CAS, and the controls by combining the significant glycans. As shown in Additional file 1: Figure S1, the significant correlation coefficients in glycans ranged from 0.145 to 0.845. There were internal associations among glycans, which could induce multicollinearity in the statistical models; therefore, the classical method of regression, including ridge, lasso, and stepwise regression, was used to select glycans to make dimension reduction [39–41]. To evaluate the performance of the discriminatory model, the 5-fold cross-validation procedure was analyzed using the *R* package “boot.” The index of evaluation was the false discrimination rates, which were used to compare the method of dimension reduction. The internal associations among glycans were analyzed using the *R* package “corrplot.” A receiver operating characteristic (ROC) curve was developed for the calculation of the area under the curve (AUC) with a 95% confidence interval (CI).

To investigate the relationship between markers of inflammation and IgG *N*-glycans, a canonical correlation analysis (CCA) was used to determine two set variables of the initial glycan structures (*x*) and markers of inflammation (*y*) and find those combinations which were maximally correlated with each other [42]. The identified variables with a statistically significant impact on the canonical variables were judged by the canonical loadings. Generally, the absolute values greater than 0.30 are considered to be significant loadings. Since normalized transformations were applied, linear correlation was used to calculate the correlation coefficient of the 24 initial glycans with markers of inflammation.

Data analysis was performed using SPSS Statistics version 21.0 for Windows (IBM Corp., Armonk, NY, USA), SAS software version 9.2 (SAS Institute, Chicago, IL, USA), and *R* version 3.3.2 (*R* Core Team 2016). All reported *p* values were two-tailed, and *p* < 0.05 was considered statistically significant.

## Results

### Participant characteristics

In total, 78 participants with IS, 75 with CAS, and 77 controls were included in the present study. The differences of demographic characteristics and inflammatory parameters among the three groups were analyzed (Table 1). The levels of BMI, SBP, CRP, TNF-α, and MMP9 in the IS group were significantly higher than those in the control group, whereas higher levels of TC and LDL were found in the control group (*p* < 0.017). The levels of CRP and MMP9 in the IS group were significantly higher than those in the CAS group, whereas higher levels of TC and LDL were found in the CAS group (*p* < 0.017).

**Table 1:** Characteristics of the study subjects

Parameters	Controls (*n* = 77)	CAS (*n* = 75)	IS (*n* = 78)	*p* value*
Gender (male/female)	54/23	54/21	60/18	0.617
Age (years)	48.09 ± 8.42	48.79 ± 7.88	47.42 ± 13.02	0.735
BMI (kg/m^2^)	23.75 ± 2.85	24.92 ± 3.17	25.65 ± 4.50^$^	0.005
FBG (mmol/L)	5.10 ± 0.52	5.14 ± 0.48	5.47 ± 2.19	0.169
SBP (mmHg)	119.35 ± 9.54	120.21 ± 9.65	124.86 ± 12.48^$^	0.002
DBP (mmHg)	76.10 ± 8.43	78.52 ± 7.78	78.68 ± 8.45	0.115
TC (mmol/L)	4.49 ± 0.73	4.74 ± 0.84	3.84 ± 1.02^$&^	< 0.001
TG (mmol/L)	1.49 ± 0.73	1.57 ± 0.96	1.70 ± 0.83	0.372
HDL (mmol/L)	1.22 ± 0.37	1.32 ± 0.31	1.09 ± 0.37^&^	0.048
LDL (mmol/L)	2.52 ± 0.50	2.68 ± 0.56	2.26 ± 0.85^$^	< 0.001
CRP (mg/L)	0.34 (0.22–0.57)	0.44 (0.22–0.86)	0.51 (0.27–1.73)^$&^	< 0.001
TNF-α (ng/L)	12.06 (8.38–17.32)	11.63 (8.21–17.55)	18.52 (12.60–24.26)^$^	0.041
MMP9 (mg/L)	0.34 (0.20–0.51)	0.23 (0.16–0.40)	0.87 (0.46–1.15)^$&^	< 0.001

*CAS* cerebral arterial stenosis, *IS* ischemic stroke, *BMI* body mass index, *FBG* fasting blood glucose, *SBP* systolic blood pressure, *DBP* diastolic blood pressure, *TC* total cholesterol, *TG* total triglycerides, *HDL* high-density lipoprotein, *LDL* low-density lipoprotein, *CRP* C-reactive protein, *TNF-α* tumor necrosis factor-alpha, *MMP9* matrix metalloproteinase-9

*Statistically significant at a significant level of 0.05

^#^*p* < 0.017, CAS group compared with control group

^$^*p* < 0.017, IS group compared with control group

^&^*p* < 0.017, IS group compared with CAS group

### Association of IgG *N*-glycans with ischemic stroke

**Fig. 1:**
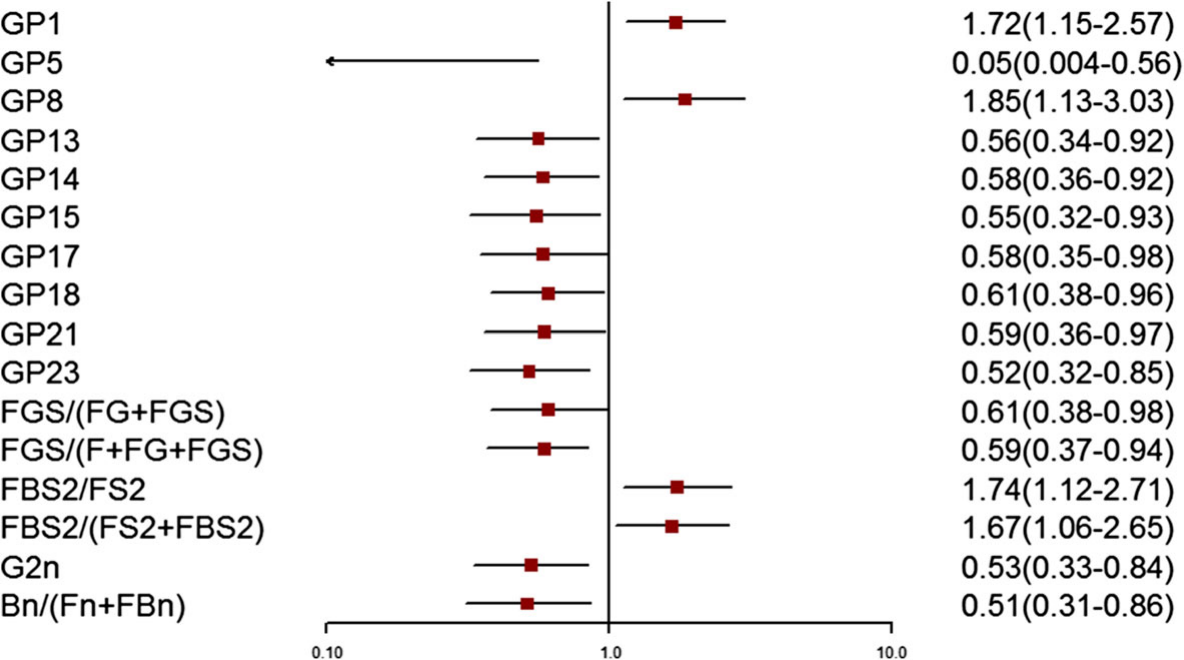
Odds ratios (OR) and 95% confidence intervals (95% CI) for the associations of the normalized glycan variables in IS versus the control (adjusted for age, sex, obesity, diabetes, hypertension, and dyslipidemia)

**Fig. 2:**
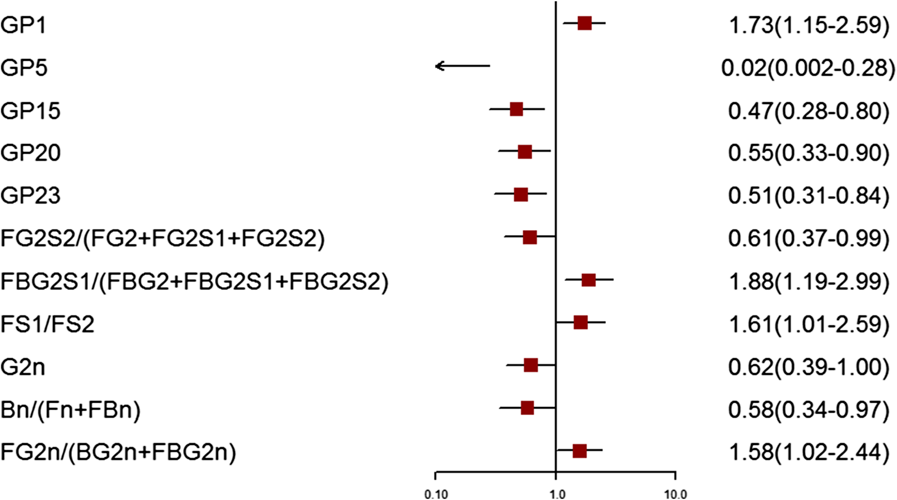
Odds ratios (OR) and 95% confidence intervals (95% CI) for the associations of the normalized glycan variables in IS versus CAS (adjusted for age, sex, obesity, diabetes, hypertension, and dyslipidemia)

Of the 24 initial glycans, 12 were abnormally distributed (*p <* 0.10) (Additional file 1: Table S1). Normalized transformations were then applied. Statistically significant differences in IgG glycome composition between patients with IS compared with CAS and controls were observed (Figs. 1 and 2, Additional file 1: Tables S2–S5), while there was no significant difference between CAS and controls (*p* > 0.05). In total, 10 initial glycans and six derived glycans (glycosylation features of galactosylation, sialylation, and bisecting GlcNAc) were different between the IS and control groups after adjusting for the effects of gender, age, obesity, diabetes, hypertension, and dyslipidemia (*p* < 0.05). Meanwhile, five initial glycans and six derived glycans (glycosylation features of galactosylation and sialylation) were different between the IS and CAS groups after adjusting for the effects of gender, age, obesity, diabetes, hypertension, and dyslipidemia (*p* < 0.05).

For the initial glycans, the levels of GP1 and GP8 in the IS group were significantly higher than those in the control group, whereas higher levels of GP5, GP13, GP14, GP15, GP17, GP18, GP21, and GP23 were found in the control group. For the derived glycans, the level of bisecting GlcNAc (FBS2/FS2 and FBS2/(FS2 + FBS2)) in the IS group was significantly higher than that in the control group, whereas higher levels of galactosylation (G2^n^) and sialylation (FGS/(FG + FGS) and FGS/(F + FG + FGS)) were found in the control group.

Among the initial glycans, the levels of GP1 in the IS group was significantly higher than those in the CAS group, whereas higher levels of GP5, GP15, GP20 and GP23 were found in the CAS group. For the derived glycans, the level of galactosylation (G2^n^) and sialylation (FG2S2/(FG2 + FG2S1 + FG2S2) and FBG2S1/(FBG2 + FBG2S1 + FBG2S2)) in the IS group were significantly lower than those in the CAS group.

### Classification of ischemic stroke using IgG glycans

**Fig. 3:**
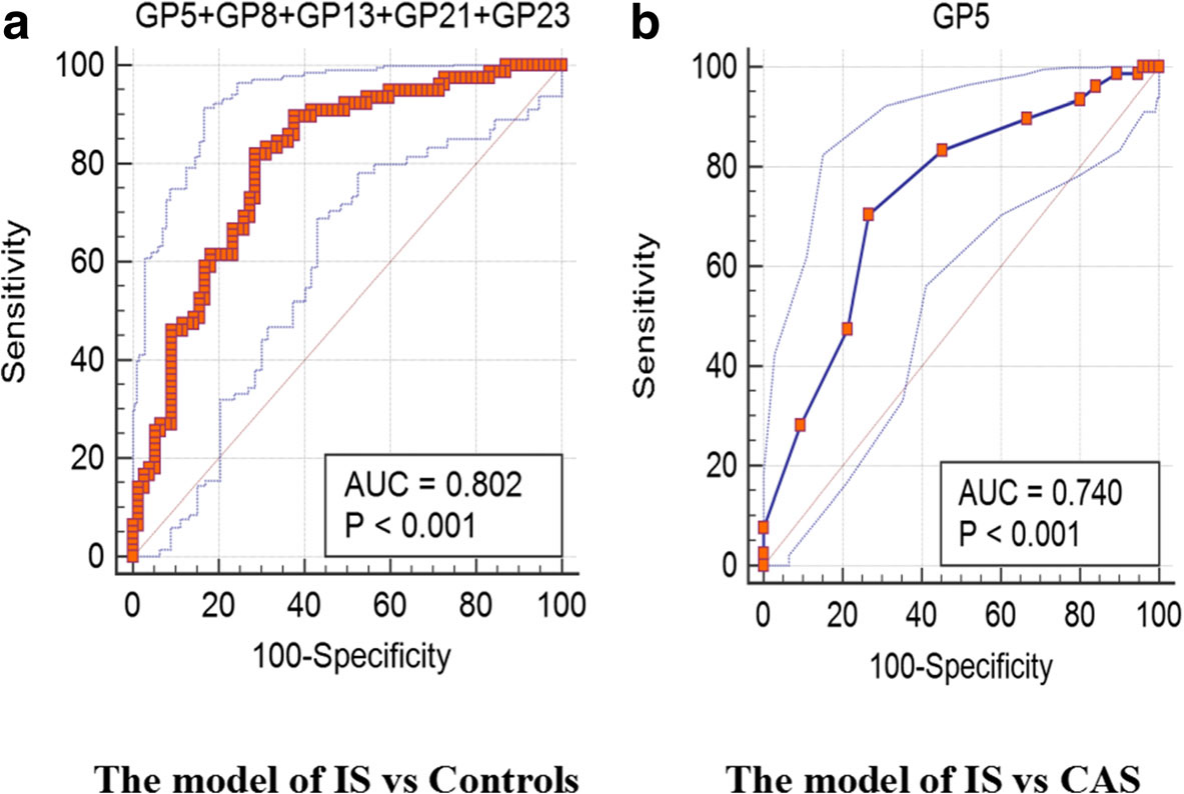
Receiver operating characteristic (ROC) curve analysis in regard to binary logistic regression in the prediction of IS by the control and CAS

GP5, GP13, and GP23 were selected by ridge regression; GP5, GP8, GP13, GP21, and GP23 were selected by step regression; and GP1, GP5, GP8, GP13, GP17, GP21, and GP23 were selected by lasso regression in the IS and control groups. The three methods of the false discrimination rates were 0.296, 0.304, and 0.272, respectively (Additional file 1: Table S6). In the end, we chose step regression to establish logistic regression models incorporating the included glycans to distinguish IS from controls, with AUC of 0.802 (95% CI, 0.732–0.872) (Fig. 3). GP5 was selected by step regression in the IS and CAS groups, with an AUC of 0.740 (95% CI, 0.661–0.819) (Fig. 3).

### Correlation between IgG glycosylation and inflammation markers

**Fig. 4:**
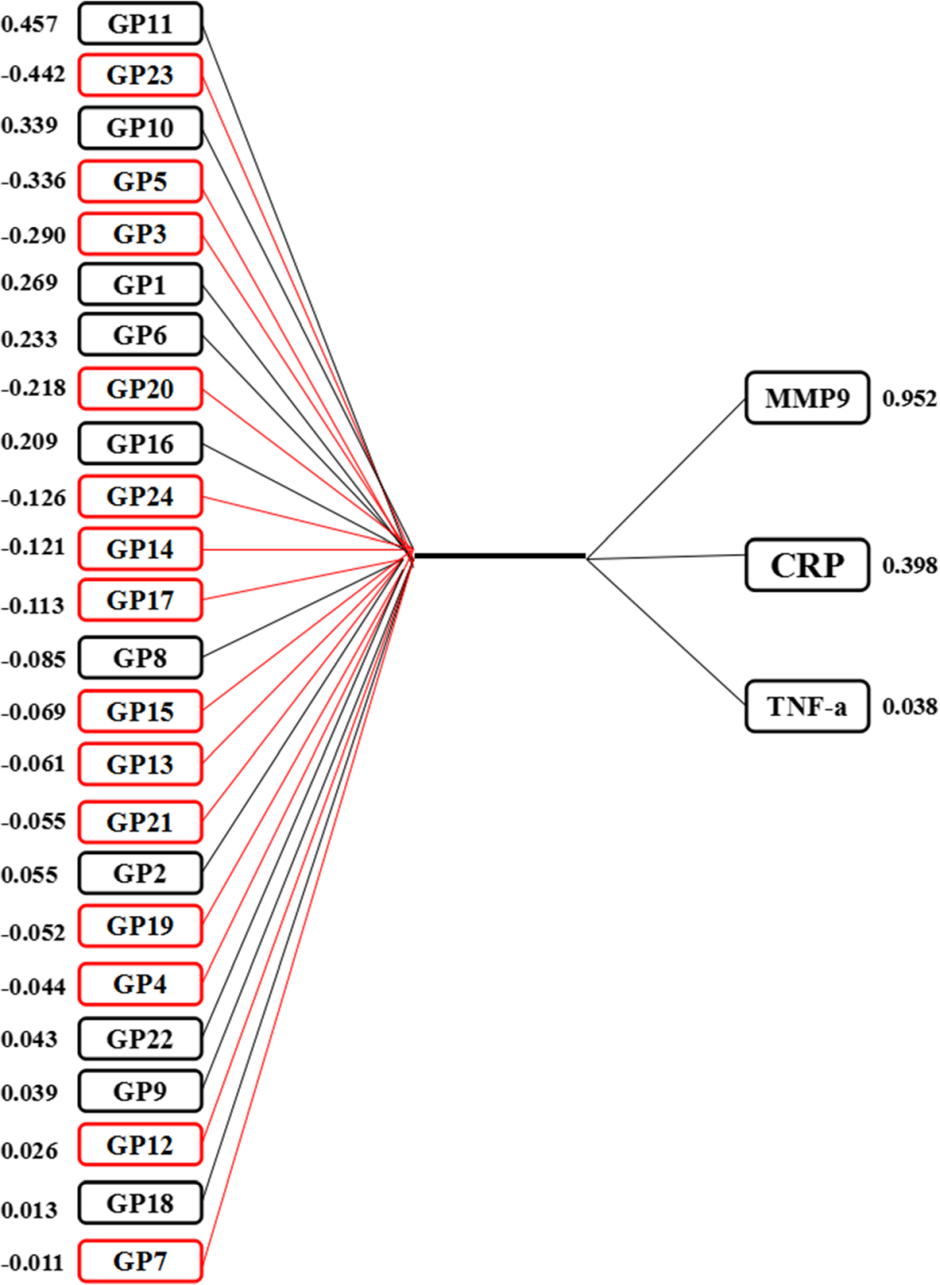
Canonical structures of initial IgG *N*-glycan and inflammation markers in the first canonical set. The absolute value of canonical loadings greater than 0.30 was significant loadings. All of the variables are sorted by the absolute value of their canonical loadings. The positive relationships are represented in black boxes, while negative relationships are showed in red boxes. CRP C-reactive protein, TNF-α tumor necrosis factor-alpha, MMP9 matrix metalloproteinase-9

The results of CCA showed that there were three pairs of canonical variables, with canonical correlations of 0.566 (*F* = 1.83, *p* < 0.001), 0.387 (*F* = 1.01, *p* = 0.467), and 0.243 (*F* = 0.56, *p* = 0.944) for each successive pair by the CCA. The first canonical set was statistically significant, indicating that *N*-glycan structures were significantly correlated with the markers of inflammation. Moreover, four initial traits (GP5, GP10, GP11, and GP23) tended to be significantly associated with MMP9 and CRP levels in the first canonical set (Fig. 4). In addition, the level of GP11 was strongly associated with canonical variables with the loading of 0.457, while the response variable with the highest canonical loading was 0.952 (MMP9).

In addition, the Pearson product moment correlation coefficients between *N*-glycan structures and inflammation markers are shown in Table 2. The inflammatory state was positively correlated with GP4, GP6, GP10, and GP11, while negatively correlated with GP5, GP14, GP18, and GP23. The association between derived glycans and markers of inflammation showed that low levels of galactosylation and sialylation and high levels of bisecting GlcNAc correspond with the state of inflammation (Additional file 1: Table S7).

**Table 2:** Associations between normalized initial glycans and inflammation markers

Glycans	CRP	TNF-α	MMP9	Inflammation
GP1	− 0.024	0.074	0.177**	+&
GP2	0.202**	0.071	0.039	+&
GP3	0.070	0.155*	− 0.141*	/
GP4	0.144*	0.185**	0.013	+#
GP5	− 0.187**	− 0.138*	− 0.219**	−#
GP6	0.202**	0.103	0.170**	+#
GP7	0.038	− 0.057	− 0.042	/
GP8	0.106	0.065	0.067	/
GP9	0.009	0.005	− 0.006	/
GP10	0.146*	− 0.008	0.198**	+#
GP11	0.178**	0.008	0.244**	+#
GP12	− 0.073	− 0.186**	− 0.051	/
GP13	− 0.014	− 0.046	− 0.065	−&
GP14	− 0.216**	− 0.198**	− 0.094	−#
GP15	− 0.072	− 0.159*	− 0.069	−&
GP16	0.073	0.017	0.108	/
GP17	− 0.084	− 0.110	− 0.077	/
GP18	− 0.156*	− 0.165*	− 0.036	−#
GP19	− 0.045	0.053	− 0.012	/
GP20	− 0.076	0.034	− 0.129	/
GP21	− 0.139*	− 0.057	− 0.025	/
GP22	0.012	0.025	0.044	/
GP23	− 0.201**	− 0.038	− 0.215**	−#
GP24	− 0.021	0.074	− 0.017	/

# there are more than two significant and consistent inflammation markers. & the result is a combination of previous studies. + the association between glycans and inflammation is positive. − the association between glycans and inflammation is negative. / the association between glycans and inflammation remains unclear. *CRP* C-reactive protein, *TNF-α* tumor necrosis factor-alpha, *MMP9* matrix metalloproteinase-9

*Correlation is significant at the 0.05 level

**Correlation is significant at the 0.01 level

## Discussion

Previous studies showed that IgG glycosylation is associated with the risk factors of IS, such as aging, obesity, dyslipidemia, T2D, and hypertension [25–28]. In this study, we described the differences of glycosylation of IgG in IS patients and compared these with CAS and the controls. To our knowledge, this study is the first attempt to investigate the association of IS with IgG *N*-glycans.

We showed the high levels of GP1 and GP8 and low levels of GP5, GP13, GP14, GP15, GP17, GP18, GP21, and GP23 in IS patients compared with the controls. GP1 and GP8 are agalactosylated and fucosylated glycans, which are significantly higher in IS. In parallel, GP13, GP14, and GP15, which have two galactoses and share the derived trait G2^n^, are significantly lower in IS. In addition, GP17, GP18, GP21, and GP23 that contain sialic acid are lower in IS. The changes of galactosylation and sialylation for initial glycans are consistent with the results of the association between the derived traits in the IgG glycome and IS. The changes of derived glycans indicate that the high level of bisecting GlcNAc can increase the risk of IS. Although we did not identify the differences of glycans between CAS and controls, the differences of glycans in IS compared with CAS or the controls were identified. The changes of glycans between IS and CAS were less compared to the changes between IS and controls, suggesting that there were some changes of glycosylation in CAS compared with controls. In the progression of IS, the faintly aberrant IgG glycosylation in CAS might play a cascading role in the pathogenesis of IS. Our findings also indicate that the high level of bisecting GlcNAc and the low level of galactosylation and sialylation may increase the risk of IS compared with CAS and controls.

Abundant evidence has shown that the decreasing galactosylation and sialylation and the increasing bisecting GlcNAc are risk factors of many inflammatory and chronic diseases [18–21, 25, 27, 28, 43, 44], which is consistent with our present results (Table 3). As summarized in Table 3, the decreased IgG galactosylation, which was reported in a number of different inflammatory and chronic diseases, might suggest that aberrant glycosylation of IgG is not disease-specific, but a general phenomenon associated with reducing the anti-inflammatory function of circulating IgG. Studies have shown that the absence of sialic acid dramatically changes the physiological role of IgG, converting them from anti-inflammatory to pro-inflammatory agents [45]. So far, this evidence clearly shows that IgG glycosylation plays a crucial role in modulating the antibody-mediated response and could be a part of the molecular mechanism leading to the promotion of inflammation. In the present study, the association between IgG glycosylation and inflammatory status showed that low levels of galactosylation and sialylation and high levels of bisecting GlcNAc correspond with the state of inflammation.

**Table 3:** The change of glycans in diseases by UPLC

Glycans	CRC	UC	CD	SLE	RA	CKD	PD	HT	T2D	O	DL*	IS
GP1	↑	–	–	↑	↑	–	–	–	–	–	–	↑
GP2	↑	–	–	↑	↑	↑	–	–	–	–	↑	–
GP3	↑	–	–	–	–	–	–	–	–	–	–	–
GP4	↑	↑	↑	↑	–	–	–	↑	–	–	↑	–
GP5	↑	–	–	↑	–	–	↓	–	–	–	↑	↓
GP6	↑	↑	↑	↑	–	↑	–	↑	↑	–	↑	–
GP7	–	–	–	↑	–	–	–	–	–	–	–	–
GP8	↓	–	–	↓	–	–	↑	–	↓	–	–	↑
GP9	↓	↓	↓	↓	–	–	–	–	↓	–	–	–
GP10	↓	–	–	↑	–	–	–	–	↑	–	–	–
GP11	↓	–	–	–	–	–	–	–	↑	–	↑	–
GP12	↓	–	–	↑	–	–	–	↓	–	–	–	–
GP13	↓	–	–	–	–	–	–	↓	–	–	–	↓
GP14	↓	↓	↓	↓	–	↓	↑	↓	–	↓	↓	↓
GP15	↓	–	–	–	–	–	–	↓	–	–	–	↓
GP16	↑	–	–	↓	–	–	–	–	–	–	–	–
GP17	–	–	–	↑	–	–	↓	–	–	–	–	↓
GP18	↓	↓	↓	↓	–	↓	–	↓	–	↓	↓	↓
GP19	↓	–	↓	↑	–	–	–	–	–	–	–	–
GP20	–	–	–	–	–	–	↓	–	–	–	↑	–
GP21	–	–	–	–	–	–	↓	–	–	–	↑	↓
GP22	↓	–	–	↑	↓	–	↓	–	–	–	–	–
GP23	–	–	–	↓	–	–	–	–	–	–	–	↓
GP24	–	–	–	↑	↑	–	–	–	–	–	–	–
Galactosylation	↓	↓	↓	↓	↓	↓	↓	↓	↓	↓	↓	↓
Sialylation	↓	–	↓	↓	–	↓	↓	↓	↓	–	↓	↓
Fucosylation	↑	–	–	↓	↑	–	–	↓	↓	–	–	–
Bisecting GlcNAc	↓	–	↑	↑	–	↑	–	↑	↑	–	↑	↑

↑ high level of glycans can increase the risk of disease. ↓ low level of glycans can increase the risk of disease. *CRC* colorectal cancer, *UC* ulcerative colitis, *CD* Crohn’s disease, *SLE* systemic lupus erythematosus, *RA* rheumatoid arthritis, *CKD* chronic kidney disease, *PD* Parkinson’s disease, *HT* hypertension, *T2D* type 2 diabetes, *O* obesity, *DL* dyslipidemia, *IS* ischemic stroke

*Unpublished paper

The high levels of pro-inflammatory parameters (CRP, TNF-α, and MMP9) in the IS group show that inflammation might be one of the characteristics of IS. This is supported by the fact that a chronic level of inflammation is a known risk factor for IS [7]. Therefore, our finding that IS is associated with the inflammatory status of IgG (lower of galactosylation and sialylation and higher of bisecting GlcNAc) might partly explain the inflammation accompanying the development of IS.

The emergence and development of molecular markers opens the “black box” of disease development. *N*-glycosylation of human proteins is an essential posttranslational modification, which is closely related to biological function, and may predict the occurrence and development of diseases more accurately [10, 46]. However, there are many challenges to transfer glycosylation biomarkers into clinical applications. As we have shown (Table 3), the change of glycans (GP5, GP8, GP17, and GP21) in several chronic diseases is not consistent. To select biomarkers for a disease screen, we first chose qualitative biomarkers to determine what conditions can increase the risk of disease. In combination with previous studies (Tables 2 and 3), we found that high level of GP1, GP2, GP4, and GP6 and low level of GP13, GP14, GP15, GP18, and GP23 can increase the risk of disease. Therefore, GP1-2, GP4, GP6, GP13-15, GP18, and GP23 may be developed as clinically useful biomarkers for chronic disease in the future.

In the present study, however, it is worth mentioning GP5 which was incorporated into the two final discriminatory models. In addition, only GP5 was used to discriminate IS from CAS with AUC 0.740. From the results of associations between initial glycans and markers of inflammation, GP5 had a strong negative association with inflammation. Based on these results, low levels of GP5 can increase the risk of IS, which is consistent with the association with Parkinson’s disease but contrary to the association with colorectal cancer, systemic lupus erythematosus, and dyslipidemia [18, 43]. A possible explanation for this phenomenon could be that IS and Parkinson’s are diseases of the nervous system and have a common pathological basis of cerebral arteriosclerosis [47–49]. In fact, GP5 is not only an agalactosylated glycan but also a high-mannose *N*-linked glycan. Studies have shown that high mannose can inhibit the inflammatory effect of macrophages and regulate the immune system to exert anti-inflammatory effects, while decrease in mannose levels can promote inflammatory effects [50]. In our present study, GP5 may be a special biomarker for the prevention or intervention of IS.

Although the inflammatory role of IgG *N*-glycosylation associates with the increased risk of ischemic stroke, this novel biomarker needs further validation in multiple-ethnic population and the development of standard operating procedure before the practical utility in screening or diagnosis of this disease. Besides the genome, epigenome, transcriptome, proteome, and metabolome, the glycome would supply new alternative for the screening of the biomarkers. However, there are several common limitations and insufficiencies, which should be recognized. First, the differential glycosylation described above may provide exciting insights into the pathogenesis of ischemic stroke. However, causation is difficult to infer in data from those already diagnosed with the condition, and the observed changes may be consequences rather than causes of the disease. Second, based on the fact that our study is a case-control study, the selection bias cannot be ignored, which could possibly lead to over-estimations of diagnostic accuracy compared with a cross-sectional study or cohort study. In addition, the exclusion criteria with history of medications during the preceding 2 weeks are limited to the controls but do not require the cases, which may trigger the bias in estimation of measuring parameters. Also, only three pro-inflammatory parameters (CRP, TNF-α, and MMP9) were examined to represent inflammatory status, which may induce evaluation bias. Finally, a possible explanation for a failure to demonstrate the differences of glycans between CAS and controls could be that the sample size is too small to identify the differences. Therefore, further cross-sectional studies and cohort studies with large samples are needed for the identification of diagnostic biomarkers for ischemic stroke and a more definite explanation about the relationships between *N*-glycan structures and ischemic stroke based on our observation.

## Conclusion

The present study showed that losses of galactose and sialic acid, as well as bisecting GlcNAc of IgG, might involve in the molecular mechanism of inflammation, which might be related to chronic inflammation that accompanies the development of IS. Aberrant IgG glycosylation might contribute to the pathogenesis of IS via loss of pro- and anti-inflammatory balance, and thus, IgG glycan profiles may be potential biomarkers for the screening or risk stratification of IS.

## Additional file

Additional file 1: **Figure S1.** The correlation coefficients in independent variables. Statistically significant associations between two variables are shown, *p* < 0.05, while the insignificant correlation coefficients are blank in the boxes. The positive correlations are represented by blue color, while the negative correlations are represented by red color. **Table S1.** Description of the IgG glycome. **Table S2.** The levels of initial glycans from controls and CAS and IS patients. **Table S3.** Associations of the normalized initial glycans (adjusted for age, sex, obesity, diabetes, hypertension, and dyslipidemia). **Table S4.** The levels of derived glycans from controls and CAS and IS patients. **Table S5.** Associations of the normalized initial glycans (adjusted for age, sex, obesity, diabetes, hypertension, and dyslipidemia). **Table S6.** The false discrimination rates of 5-fold cross-validation in the three methods. Table S7 Associations between derived glycans and inflammation markers. (DOC 691 kb)

## Abbreviations

ANOVA: Analysis of variance
AUC: Area under the curve
BMI: Body mass index
CAS: Cerebral arterial stenosis
CCA: Canonical correlation analysis
CI: Confidence intervals
CRP: C-reactive protein
CT: Computed tomography
DBP: Diastolic blood pressure
ELISA: Enzyme-linked immunosorbent assay
FBG: Fasting blood glucose
Fc: Fragment crystallizable
GP: Glycan peak
GPs: Glycan peaks
HDL: High-density lipoprotein
IgG: Immunoglobulin G
IS: Ischemic stroke
LDL: Low-density lipoprotein
MMP9: Matrix metalloproteinase-9
MRI: Magnetic resonance imaging
OR: Odds ratio
ROC: Receiver operating characteristic
SBP: Systolic blood pressure
SD: Standard deviation
T2D: Type 2 diabetes
TC: Total cholesterol
TG: Triglycerides
TNF-α: Tumor necrosis factor-alpha
UPLC: Ultra-performance liquid chromatography
WHO: World Health Organization
