# Supplementary material for: Ischemic stroke is associated with the pro-inflammatory potential of N-glycosylated immunoglobulin G
Source: J Neuroinflammation. 2018 Apr 26;15:123. doi: 10.1186/s12974-018-1161-1 (PMC5921323; doi:10.1186/s12974-018-1161-1)
Supplement: Supplementary file 1 — Figure S1. The correlation coefficients in independent variables. Statistically significant associations between two variables are shown, p < 0.05, while the insignificant correlation coefficients are blank in the boxes. The positive correlations are represented by blue color, while the negative correlations are represented by red color. Table S1. Description of the IgG glycome. Table S2. The levels of initial glycans from controls and CAS and IS patients. Table S3. Associations of the normalized initial glycans (adjusted for age, sex, obesity, diabetes, hypertension, and dyslipidemia). Table S4. The levels of derived glycans from controls and CAS and IS patients. Table S5. Associations of the normalized initial glycans (adjusted for age, sex, obesity, diabetes, hypertension, and dyslipidemia). Table S6. The false discrimination rates of 5-fold cross-validation in the three methods. Table S7 Associations between derived glycans and inflammation markers. (DOC 691 kb) [file 12974_2018_1161_MOESM1_ESM.doc]

Additional file 1


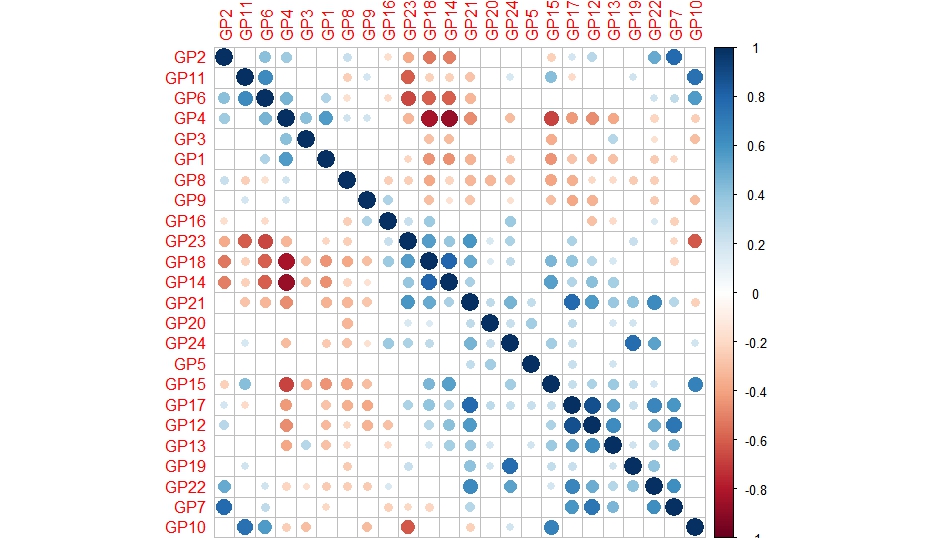


Figure S1. The correlation coefficients in independent variables.

Statistically significant associations between two variables are shown, *p*<0.05, while the insignificant correlation coefficients are blank in the boxes. The positive correlations are represented by blue color, while negative correlations are represented by red color.

Table S1 Description of the IgG glycome

| Glycan peak | Peak composition | Medians (*P*_25-_ *P*_75_) | Z | *p* value*** |
| --- | --- | --- | --- | --- |
| GP1 | 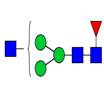 | 0.08(0.07-0.11) | 2.278 | <0.001 |
| GP2 | 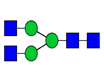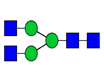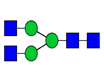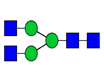 | 0.54(0.37-0.76) | 2.033 | 0.001 |
| GP3 | 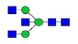 | 0.09(0.06-0.13) | 1.946 | 0.001 |
| GP4 | 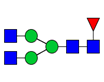 | 20.38(16.71-23.49) | 0.719 | 0.680 |
| GP5 | 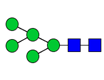 | 0.05(0.03-0.06) | 5.181 | <0.001 |
| GP6 | 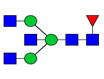 | 4.65(3.97-5.63) | 1.149 | 1.143 |
| GP7 | 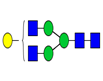 | 0.28(0.20-0.41) | 1.751 | 0.004 |
| GP8 | 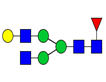 | 17.74(16.45-18.73) | 0.757 | 0.616 |
| GP9 | 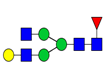 | 9.86(8.91-10.58) | 0.804 | 0.537 |
| GP10 | 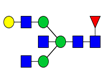 | 4.39(3.76-5.12) | 0.791 | 0.558 |
| GP11 | 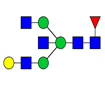 | 0.60(0.52-0.68) | 1.565 | 0.015 |
| GP12 | 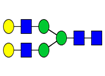 | 0.85(0.57-1.22) | 1.601 | 0.012 |
| GP13 | 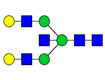 | 0.22(0.19-0.26) | 2.063 | <0.001 |
| GP14 | 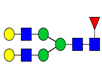 | 16.16(13.73-17.97) | 0.610 | 0.850 |
| GP15 | 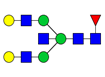 | 1.32(1.10-1.61) | 0.964 | 0.311 |
| GP16 | 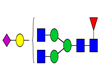 | 2.88(2.50-3.27) | 0.631 | 0.821 |
| GP17 | 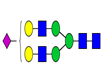 | 0.87(0.76-1.04) | 1.658 | 0.008 |
| GP18 | 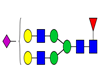 | 11.12(9.65-12.81) | 0.651 | 0.791 |
| GP19 | 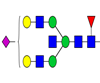 | 2.11(1.91-2.42) | 0.718 | 0.680 |
| GP20 | 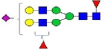 | 0.03(0.02-0.05) | 2.709 | <0.001 |
| GP21 | 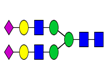 | 0.65(0.57-0.74) | 1.658 | 0.008 |
| GP22 | 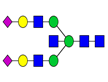 | 0.15(0.12-0.19) | 2.165 | <0.001 |
| GP23 | 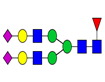 | 1.91(1.53-2.39) | 0.941 | 0.339 |
| GP24 | 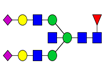 | 2.14(1.81-2.45) | 0.804 | 0.538 |

* Normality distributions of glycans were tested by the Kolmogorov-Smirnov tests, and of which *p*< 0.10 was considered statistically significant.

Table S2. The levles of initial glycans from controls, CAS and IS patients

| GPs | Controls  (n=77) | CAS  (n=75) | IS  (n=78) | *p* value*** |
| --- | --- | --- | --- | --- |
| GP1 | 0.08(0.06-0.10) | 0.08(0.06-0.10) | 0.09(0.08-0.11)^&^ | 0.014 |
| GP2 | 0.48(0.35-0.76) | 0.54(0.37-0.79) | 0.57(0.40-0.75) | 0.395 |
| GP3 | 0.09(0.07-0.13) | 0.10(0.05-0.15) | 0.09(0.05-0.12) | 0.203 |
| GP4 | 19.56(15.95-23.14) | 20.31(16.21-23.42) | 21.18(18.19-23.93) | 0.107 |
| GP5 | 0.05(0.04-0.07) | 0.06(0.04-0.07) | 0.04(0.02-0.05)^$&^ | <0.001 |
| GP6 | 4.48(3.93-5.35) | 4.58(3.92-5.56) | 5.00(4.26-5.85)^$^ | 0.028 |
| GP7 | 0.27(0.22-0.41) | 0.29(0.22-0.43) | 0.28(0.17-0.40) | 0.284 |
| GP8 | 17.08(16.15-18.31) | 17.76(16.41-18.75) | 18.23(16.94-19.02)^$^ | 0.004 |
| GP9 | 9.88(8.90-10.64) | 9.86(8.51-10.63) | 9.86(8.96-10.41) | 0.908 |
| GP10 | 4.13(3.57-4.90) | 4.26(3.78-5.11) | 4.64(3.96-5.62) | 0.011 |
| GP11 | 0.58(0.51-0.65) | 0.60(0.51-0.66) | 0.63(0.55-0.72)^$^ | 0.016 |
| GP12 | 0.92(0.69-1.33) | 0.88(0.56-1.28) | 0.75(0.48-1.15)^$^ | 0.031 |
| GP13 | 0.23(0.21-0.27) | 0.23(0.19-0.28) | 0.21(0.18-0.24)^$&^ | 0.007 |
| GP14 | 16.56(14.20-18.59) | 16.23(13.52-18.26) | 15.60(13.18-17.26) | 0.110 |
| GP15 | 1.35(1.12-1.63) | 1.37(1.12-1.69) | 1.22(1.04-1.53) | 0.086 |
| GP16 | 2.84(2.56-3.30) | 2.96(2.46-3.40) | 2.86(2.45-3.22) | 0.691 |
| GP17 | 0.92(0.82-1.11) | 0.88(0.78-1.09) | 0.83(0.70-0.94)^$&^ | 0.001 |
| GP18 | 11.69(9.84-13.60) | 11.26(10.12-12.55) | 10.74(0.93-12.25)^$^ | 0.026 |
| GP19 | 2.10(1.93-2.41) | 2.17(1.92-2.39) | 2.11(1.81-2.47) | 0.952 |
| GP20 | 0.04(0.02-0.06) | 0.04(0.02-0.06) | 0.03(0.02-0.04)^$&^ | <0.001 |
| GP21 | 0.66(0.59-0.80) | 0.66(0.58-0.77) | 0.63(0.53-0.70)^$^ | 0.024 |
| GP22 | 0.16(0.12-0.20) | 0.15(0.12-0.20) | 0.15(0.10-0.19) | 0.454 |
| GP23 | 2.03(1.72-2.57) | 2.13(1.67-2.51) | 1.69(1.33-2.11)^$&^ | <0.001 |
| GP24 | 2.14(1.82-2.43) | 2.20(1.91-2.56) | 1.99(1.75-2.45) | 0.221 |

* Statistically significant at significant level of 0.05.

# *p* < 0.017, CAS group compared with control group.

$ *p* < 0.017, IS group compared with control group.

& *p* < 0.017, IS group compared with CAS group.

CAS:Cerebral arterial stenosis; IS: ischemic stroke.

Table S3. Associations of the normalized initial glycans (adjusted for age, sex, obesity, diabetes, hypertension, dyslipidemia)

| **GPs** | **CAS vs controls** | | **IS vs controls** | | **IS vs CAS** | |
| --- | --- | --- | --- | --- | --- | --- |
|  | OR (95% CI) | *p** | OR (95% CI)^a^ | *p** | OR (95% CI)^a^ | *p** |
| GP1 | 0.99(0.71-1.39) | 0.964 | 1.72(1.15-2.57) | 0.009 | 1.73(1.15-2.59) | 0.008 |
| GP2 | 1.17(0.80-1.70) | 0.417 | 1.34(0.84-2.13) | 0.222 | 1.14(1.73-1.79) | 0.558 |
| GP3 | 1.09(0.80-1.48) | 0.598 | 0.93(0.60-1.45) | 0.761 | 0.86(0.56-1.32) | 0.494 |
| GP4 | 1.05(0.75-1.49) | 0.772 | 1.53(0.98-2.37) | 0.061 | 1.45(0.93-2.26) | 0.102 |
| GP5 | 2.09(0.44-9.85) | 0.350 | 0.05(0.004-0.56) | 0.015 | 0.02(0.002-0.28) | 0.003 |
| GP6 | 1.16(0.81,1.65) | 0.424 | 1.50(0.98,2.31) | 0.065 | 1.30(0.85-1.99) | 0.230 |
| GP7 | 1.16(0.84-1.59) | 0.369 | 0.83(0.52-1.34) | 0.448 | 0.72(0.45-1.15) | 0.170 |
| GP8 | 1.20(0.84-1.73) | 0.322 | 1.85(1.13-3.03) | 0.015 | 1.54(0.94-2.50) | 0.085 |
| GP9 | 0.93(0.68-1.26) | 0.624 | 0.99(0.66-1.48) | 0.954 | 1.07(0.71-1.61) | 0.756 |
| GP10 | 1.20(0.85-1.70) | 0.293 | 1.40(0.90-2.17) | 0.140 | 1.16(0.75-1.79) | 0.506 |
| GP11 | 1.17(0.81-1.69) | 0.404 | 1.40(0.92-2.15) | 0.121 | 1.20(0.81-1.77) | 0.358 |
| GP12 | 0.86(0.63-1.18) | 0.347 | 0.67(0.42-1.08) | 0.100 | 0.78(0.49-1.26) | 0.315 |
| GP13 | 0.90(0.66-1.23) | 0.502 | 0.56(0.34-0.92) | 0.021 | 0.62(0.37-1.02) | 0.060 |
| GP14 | 0.84(0.60-1.19) | 0.328 | 0.58(0.36-0.92) | 0.020 | 0.69(0.43-1.09) | 0.111 |
| GP15 | 1.17(0.86-1.60) | 0.326 | 0.55(0.32-0.93) | 0.024 | 0.47(0.28-0.80) | 0.005 |
| GP16 | 1.03(0.74-1.42) | 0.872 | 0.95(0.62-1.45) | 0.814 | 0.93(0.61-1.42) | 0.723 |
| GP17 | 0.89(0.64-1.23) | 0.465 | 0.58(0.35-0.98) | 0.040 | 0.66(0.39-1.11) | 0.114 |
| GP18 | 0.83(0.59-1.17) | 0.293 | 0.61(0.38-0.96) | 0.034 | 0.73(0.46-1.16) | 0.180 |
| GP19 | 1.08(0.77-1.52) | 0.662 | 1.05(0.67-1.66) | 0.826 | 0.98(0.61-1.55) | 0.917 |
| GP20 | 1.15(0.84-1.57) | 0.378 | 0.63(0.38-1.03) | 0.066 | 0.55(0.33-0.90) | 0.018 |
| GP21 | 0.89(0.65-1.22) | 0.466 | 0.59(0.36-0.97) | 0.037 | 0.67(0.41-1.10) | 0.110 |
| GP22 | 1.07(0.74-1.54) | 0.712 | 0.84(0.50-1.40) | 0.497 | 0.78(0.47-1.31) | 0.347 |
| GP23 | 1.02(0.73-1.43) | 0.913 | 0.52(0.32-0.85) | 0.009 | 0.51(0.31-0.84) | 0.008 |
| GP24 | 1.24(0.88-1.74) | 0.218 | 0.92(0.58-1.46) | 0.717 | 0.74(0.46-1.19) | 0.214 |

* Statistically significant at significant level of 0.05.

CAS:Cerebral arterial stenosis; IS: ischemic stroke.

Table S4. The levles of derived glycans from controls, CAS and IS patients

| Glycans | Normal group  (n=77) | CAS group  (n=75) | IS group  (n=78) | *p* value*** |
| --- | --- | --- | --- | --- |
| FGS/(FG+FGS) | 27.68(25.43-29.67) | 27.22(24.59-29.57) | 26.33(23.54-27.83)^$&^ | 0.018 |
| FBGS/(FBG+FBGS) | 42.09(37.52-45.13) | 40.62(36.83-45.85) | 38.55(34.32-44.01)^$^ | 0.046 |
| FGS/(F+FG+FGS) | 21.07(18.21-24.04) | 20.57(18.13-22.36) | 19.59(16.67-21.02)^$&^ | 0.010 |
| FBGS/(FB+FBG+FBGS) | 29.42(25.57-31.61) | 27.51(24.34-33.43) | 26.14(22.08-31.67)^$^ | 0.028 |
| FG1S1/(FG1+FG1S1) | 9.62(8.51-10.75) | 9.78(8.54-10.83) | 9.31(8.11-10.48)2 | 0.225 |
| FG2S1/(FG2+FG2S1+FG2S2) | 38.31(36.13-40.72) | 38.14(35.75-40.76) | 38.69(36.15-40.08) | 0.963 |
| FG2S2/(FG2+FG2S1+FG2S2) | 6.81(5.52-8.15) | 6.89(5.76-8.27) | 5.95(5.19-7.39)^$&^ | 0.003 |
| FBG2S1/(FBG2+FBG2S1+FBG2S2) | 37.77(35.16-40.90) | 37.58(34.90-39.82) | 39.38(36.31-41.27)^&^ | 0.025 |
| FBG2S2/(FBG2+FBG2S1+FBG2S2) | 37.81(35.08-40.46) | 38.46(35.46-41.33) | 37.24(34.99-40.25) | 0.405 |
| F^total^S1/F^total^S2 | 3.93(3.51-4.49) | 3.84(3.42-4.32) | 4.13(3.59-4.80)^&^ | 0.034 |
| FS1/FS2 | 6.98(5.91-8.73) | 7.02(5.94-8.66) | 8.17(7.09-9.62)^$&^ | 0.002 |
| FBS1/FBS2 | 1.02(0.91-1.12) | 0.97(0.88-1.06) | 1.03(0.94-1.14)^&^ | 0.030 |
| FBS^total^/FS^total^ | 0.25(0.21-0.31) | 0.27(0.23-0.31) | 0.28(0.22-0.33) | 0.328 |
| FBS1/FS1 | 0.14(0.12-0.18) | 0.15(0.12-0.17) | 0.16(0.12-0.19) | 0.426 |
| FBS2/FS2 | 1.04(0.82-1.29) | 1.06(0.90-1.29) | 1.21(0.98-1.54)^$&^ | 0.007 |
| FBS1/(FS1+FBS1) | 12.34(10.63-15.43) | 13.16(11.10-14.80) | 13.43(10.85-16.23) | 0.426 |
| FBS2/(FS2+FBS2) | 51.06(45.07-56.42) | 51.52(47.24-56.34) | 54.82(49.62-60.69)^$&^ | 0.007 |
| GP1^n^ | 0.11(0.08-0.13) | 0.11(0.08-0.13) | 0.12(0.10-0.14)^&^ | 0.029 |
| GP2^n^ | 0.60(0.45-0.98) | 0.73(0.45-1.01) | 0.74(0.51-0.94) | 0.481 |
| GP4^n^ | 25.25(21.78-28.70) | 25.66(21.79-29.41) | 26.44(22.87-30.36) | 0.229 |
| GP5^n^ | 0.06(0.05-0.09) | 0.08(0.05-0.09) | 0.05(0.03-0.06)^$&^ | <0.001 |
| GP6^n^ | 5.77(5.09-6.99) | 6.03(5.04-7.33) | 6.39(5.49-7.24) | 0.072 |
| GP7^n^ | 0.36(0.29-0.54) | 0.37(0.29-0.57) | 0.35(0.21-0.49) | 0.176 |
| GP8^n^ | 22.45(21.49-23.72) | 22.75(21.90-24.27) | 23.43(21.81-24.25) | 0.071 |
| GP9^n^ | 12.95(11.52-13.790 | 12.60(11.24-13.82) | 12.43(11.18-13.24) | 0.310 |
| GP10^n^ | 5.38(4.58-6.29) | 5.46(4.93-6.60) | 5.86(5.16-6.93) | 0.050 |
| GP11^n^ | 0.73(0.65-0.87) | 0.77(0.66-0.86) | 0.80(0.70-0.93) | 0.091 |
| GP12^n^ | 1.19(0.86-1.71) | 1.09(0.71-1.64) | 0.95(0.60-1.47)^$^ | 0.020 |
| GP13^n^ | 0.30(0.26-0.35) | 0.30(0.25-0.37) | 0.27(0.24-0.31)^$&^ | 0.001 |
| GP14^n^ | 21.38(17.55-24.88) | 20.97(17.58-23.72) | 19.86(16.88-22.51)^$^ | 0.049 |
| GP15^n^ | 1.75(1.44-2.21) | 1.76(1.44-2.23) | 1.58(1.28-1.98)^&^ | 0.041 |
| G0^n^ | 32.88(27.92-37.15) | 32.67(27.90-37.26) | 34.02(29.69-38.82) | 0.142 |
| G1^n^ | 42.42(40.88-43.44) | 42.57(40.73-44.26) | 42.94(41.15-44.79) | 0.222 |
| G2^n^ | 24.75(21.02-29.26) | 24.83(20.04-27.84) | 22.57(19.43-25.73)^$^ | 0.021 |
| F^ntotal^ | 97.50(96.37-97.95) | 97.57(96.34-98.19) | 97.62(96.71-98.40) | 0.264 |
| FG0^ntotal^/G0^n^ | 98.00(96.99-98.490 | 97.80(96.83-98.51) | 97.85(96.90-98.54) | 0.771 |
| FG1^ntotal^/G1^n^ | 99.11(98.71-99.32) | 99.12(98.68-99.34) | 99.19(98.85-99.52) | 0.120 |
| FG2^ntotal^/G2^n^ | 94.01(92.25-95.10) | 94.27(92.52-95.42) | 94.22(92.78-95.77) | 0.205 |
| F^n^ | 82.99(80.98-85.15) | 83.38(80.58-85.19) | 82.31(80.06-84.74) | 0.451 |
| FG0^n^/G0^n^ | 78.67(76.59-82.14) | 78.99(75.67-82.14) | 78.02(75.56-81.42) | 0.737 |
| FG1^n^/G1^n^ | 84.02(82.02-86.30) | 84.14(81.42-86.06) | 83.21(80.57-85.49) | 0.177 |
| FG2^n^/G2^n^ | 86.36(84.27-88.18) | 86.90(84.14-88.49) | 87.23(85.23-88.70) | 0.258 |
| FB^n^ | 13.98(12.45-15.52) | 14.16(12.43-16.13) | 14.91(12.82-17.29) | 0.072 |
| FBG0^n^/G0^n^ | 19.31(16.15-21.13) | 18.85(16.49-21.01) | 19.70(16.70-21.34) | 0.769 |
| FBG1^n^/G1^n^ | 15.10(12.57-16.91) | 14.94(13.08-17.39) | 15.91(1.376-18.33) | 0.087 |
| FBG2^n^/G2^n^ | 6.87(6.16-7.99) | 7.48(6.43-8.72) | 7.10(6.03-8.41) | 0.178 |
| FB^n^/F^n^ | 16.79(14.40-18.99) | 16.93(14.63-19.76) | 18.20(15.37-21.62) | 0.098 |
| F^n^/(B^n^+FB^n^) | 5.85(5.16-6.75) | 5.81(4.97-6.67) | 5.39(4.54-6.37) | 0.128 |
| B^n^/(F^n^+FB^n^) | 3.07(2.70-3.640 | 3.09(2.51-3.76) | 2.76(2.41-3.20)^$&^ | 0.002 |
| FBG2^n^/FG2^n^ | 0.08(0.07-0.09) | 0.09(0.07-0.10) | 0.08(0.07-0.10) | 0.247 |
| FG2^n^/(BG2^n^+FBG2^n^) | 10.53(8.97-11.870 | 9.97(8.53-11.61) | 10.21(9.23-12.08) | 0.277 |
| BG2^n^/(FG2^n^+FBG2^n^) | 13.06(11.15-15.66) | 13.09(11.39-15.42) | 12.67(10.99-14.78) | 0.673 |
| FB^n^/F^ntotal^ | 14.37(12.59-15.96) | 14.48(12.76-16.50) | 15.40(13.32-17.77) | 0.098 |
| FBG2^n^/(FG2^n^+FBG2^n^) | 7.43(6.61-8.55) | 7.95(6.65-9.25) | 7.58(6.41-8.84) | 0.247 |

* Statistically significant at significant level of 0.05.

# *p* < 0.017, CAS group compared with control group.

$ *p* < 0.017, IS group compared with control group.

& *p* < 0.017, IS group compared with CAS group.

CAS:Cerebral arterial stenosis; IS: ischemic stroke.

Table S5. Associations of the normalized initial glycans (adjusted for age, sex, obesity, diabetes, hypertension, dyslipidemia)

| Glycans | CAS vs controls | | IS vs controls | | IS vs CAS | | |
| --- | --- | --- | --- | --- | --- | --- | --- |
|  | OR (95% CI) | *P** | OR (95% CI) | *P** | OR (95% CI) | | *P** |
| FGS/(FG+FGS) | 0.93(0.67-1.31) | 0.683 | 0.61(0.38-0.98) | 0.043 | 0.65(0.40,1.06) | 0.083 | |
| FBGS/(FBG+FBGS) | 0.98(0.70-1.37) | 0.914 | 0.84(0.54-1.29) | 0.422 | 0.85(0.55-1.33) | 0.477 | |
| FGS/(F+FG+FGS) | 0.91(0.65-1.27) | 0.573 | 0.59(0.37-0.94) | 0.025 | 0.65(0.40-1.03) | 0.067 | |
| FBGS/(FB+FBG+FBGS) | 0.99(0.71-1.39) | 0.950 | 0.77(0.50-1.19) | 0.231 | 0.77(0.50-1.21) | 0.258 | |
| FG1S1/(FG1+FG1S1) | 0.99(0.72-1.37) | 0.961 | 0.80(0.51-1.25) | 0.327 | 0.81(0.51-1.27) | 0.349 | |
| FG2S1/(FG2+FG2S1+FG2S2) | 0.97(0.70-1.34) | 0.843 | 1.04(0.68-1.61) | 0.846 | 1.08(0.70-1.67) | 0.733 | |
| FG2S2/(FG2+FG2S1+FG2S2) | 1.15(0.84-1.58) | 0.379 | 0.70(0.43-1.14) | 0.148 | 0.61(0.37-0.99) | 0.044 | |
| FBG2S1/(FBG2+FBG2S1+FBG2S2) | 0.80(0.57-1.13) | 0.198 | 1.50(0.96-2.34) | 0.072 | 1.88(1.19-2.99) | 0.007 | |
| FBG2S2/(FBG2+FBG2S1+FBG2S2) | 1.12(0.81-1.56) | 0.490 | 1.12(0.72-1.73) | 0.621 | 1.00(0.64-1.55) | 0.981 | |
| F^total^S1/F^total^S2 | 0.77(0.52-1.14) | 0.189 | 1.14(0.73-1.80) | 0.561 | 1.48(0.92-2.40) | 0.109 | |
| FS1/FS2 | 0.89(0.61-1.29) | 0.524 | 1.43(0.91-2.26) | 0.126 | 1.61(1.01-2.59) | 0.046 | |
| FBS1/FBS2 | 0.79(0.55-1.13) | 0.200 | 1.14(0.75-1.74) | 0.545 | 1.44(0.92-2.25) | 0.108 | |
| FBS^total^/FS^total^ | 1.23(0.86-1.75) | 0.260 | 1.46(0.93-2.29) | 0.104 | 1.19(0.77-1.85) | 0.443 | |
| FBS1/FS1 | 1.14(0.81-1.62) | 0.452 | 1.37(0.88-2.13) | 0.164 | 1.20(0.77-1.86) | 0.417 | |
| FBS2/FS2 | 1.13(0.79-1.63) | 0.502 | 1.74(1.12-2.71) | 0.015 | 1.54(0.99-2.38) | 0.054 | |
| FBS1/(FS1+FBS1) | 1.15(0.81-1.62) | 0.443 | 1.36(0.87-2.13) | 0.176 | 1.19(0.76-1.85) | 0.446 | |
| FBS2/(FS2+FBS2) | 1.14(0.81-1.61) | 0.457 | 1.67(1.06-2.65) | 0.029 | 1.47(0.93-2.33) | 0.104 | |
| GP1^n^ | 0.99(0.71-1.38) | 0.951 | 1.66(1.11-2.47) | 0.013 | 1.68(1.12-2.50) | 0.011 | |
| GP2^n^ | 1.19(0.82-1.73) | 0.374 | 1.30(0.81-2.07) | 0.277 | 1.09(0.70-1.71) | 0.696 | |
| GP4^n^ | 1.05(0.75-1.48) | 0.766 | 1.47(0.95-2.27) | 0.087 | 1.39(0.90-2.17) | 0.142 | |
| GP5^n^ | 1.98(0.43-9.17) | 0.380 | 0.03(0.003-0.42) | 0.008 | 0.02(0.001-0.22) | 0.002 | |
| GP6^n^ | 1.16(0.82-1.65) | 0.411 | 1.43(0.93-2.20) | 0.106 | 1.23(0.80-1.89) | 0.340 | |
| GP7^n^ | 1.16(0.85-1.59) | 0.358 | 0.78(0.48-1.27) | 0.320 | 0.67(0.41-1.10) | 0.110 | |
| GP8^n^ | 1.15(0.82-1.63) | 0.417 | 1.35(0.86-2.12) | 0.188 | 1.17(0.75-1.84) | 0.489 | |
| GP9^n^ | 0.90(0.66-1.24) | 0.527 | 0.85(0.56-1.27) | 0.422 | 0.94(0.62-1.42) | 0.752 | |
| GP10^n^ | 1.18(0.84-1.65) | 0.344 | 1.26(0.81-1.94) | 0.307 | 1.07(0.69-1.64) | 0.768 | |
| GP11^n^ | 1.14(0.80-1.63) | 0.456 | 1.29(0.85-1.96) | 0.240 | 1.13(0.76-1.66) | 0.550 | |
| GP12^n^ | 0.86(0.63-1.18) | 0.339 | 0.64(0.40-1.04) | 0.073 | 0.75(0.46-1.22) | 0.246 | |
| GP13^n^ | 0.89(0.65-1.20) | 0.440 | 0.51(0.31-0.85) | 0.010 | 0.58(0.35-0.97) | 0.036 | |
| GP14^n^ | 0.83(0.59-1.17) | 0.288 | 0.55(0.34-0.88) | 0.012 | 0.66(0.42-1.06) | 0.086 | |
| GP15^n^ | 1.14(0.83-1.55) | 0.418 | 0.51(0.30-0.87) | 0.014 | 0.45(0.26-0.77) | 0.004 | |
| G0^n^ | 1.09(0.78-1.54) | 0.612 | 1.54(0.99-2.40) | 0.053 | 1.41(0.91-2.20) | 0.125 | |
| G1^n^ | 1.17(0.83-1.63) | 0.374 | 1.29(0.81-2.04) | 0.279 | 1.11(0.69-1.76) | 0.672 | |
| G2^n^ | 0.84(0.60-1.19) | 0.332 | 0.53(0.33-0.84) | 0.008 | 0.62(0.39-1.00) | 0.049 | |
| F^ntotal^ | 1.02(0.74-1.41) | 0.890 | 1.27(0.80-2.03) | 0.306 | 1.25(0.78-1.99) | 0.355 | |
| FG0^ntotal^/G0^n^ | 0.85(0.60-1.20) | 0.353 | 0.88(0.55-1.39) | 0.578 | 1.04(0.66-1.63) | 0.881 | |
| FG1^ntotal^/G1^n^ | 0.89(0.65-1.21) | 0.449 | 1.33(0.81-2.19) | 0.263 | 1.50(0.91-2.47) | 0.109 | |
| FG2^ntotal^/G2^n^ | 1.14(0.83-1.56) | 0.433 | 1.23(0.80-1.89) | 0.354 | 1.08(0.70-1.68) | 0.728 | |
| F^n^ | 0.87(0.63-1.20) | 0.394 | 0.91(0.60-1.39) | 0.664 | 1.05(0.69-1.61) | 0.824 | |
| FG0^n^/G0^n^ | 0.92(0.66-1.27) | 0.601 | 0.99(0.65-1.52) | 0.975 | 1.08(0.70-1.67) | 0.715 | |
| FG1^n^/G1^n^ | 0.86(0.62-1.20) | 0.379 | 0.88(0.57-1.35) | 0.552 | 1.02(0.66-1.56) | 0.939 | |
| FG2^n^/G2^n^ | 0.94(0.69-1.29) | 0.704 | 1.25(0.80-1.94) | 0.335 | 1.32(0.85-2.07) | 0.219 | |
| FB^n^ | 1.21(0.86-1.69) | 0.277 | 1.24(0.80-1.92) | 0.334 | 1.03(0.67-1.59) | 0.902 | |
| FBG0^n^/G0^n^ | 1.05(0.76-1.47) | 0.758 | 0.97(0.63-1.49) | 0.885 | 0.92(0.59-1.42) | 0.706 | |
| FBG1^n^/G1^n^ | 1.14(0.82-1.59) | 0.426 | 1.19(0.77-1.82) | 0.432 | 1.04(0.68-1.59) | 0.862 | |
| FBG2^n^/G2^n^ | 1.35(0.96-1.91) | 0.084 | 0.87(0.53-1.43) | 0.574 | 0.64(0.39-1.06) | 0.082 | |
| FB^n^/F^n^ | 1.22(0.87-1.70) | 0.255 | 1.22(0.80-1.89) | 0.360 | 1.01(0.66-1.55) | 0.972 | |
| F^n^/(B^n^+FB^n^) | 0.87(0.61-1.25) | 0.454 | 0.88(0.56-1.38) | 0.570 | 1.01(0.63-1.60) | 0.977 | |
| B^n^/(F^n^+FB^n^) | 0.89(0.65-1.21) | 0.446 | 0.51(0.31-0.86) | 0.011 | 0.58(0.34-0.97) | 0.037 | |
| FBG2^n^/FG2^n^ | 1.34(0.95-1.89) | 0.097 | 0.86(0.52-1.44) | 0.566 | 0.64(0.38-1.07) | 0.091 | |
| FG2^n^/(BG2^n^+FBG2^n^) | 0.81(0.57-1.14) | 0.217 | 1.27(0.84-1.94) | 0.260 | 1.58(1.02-2.44) | 0.041 | |
| BG2^n^/(FG2^n^+FBG2^n^) | 0.97(0.70-1.36) | 0.868 | 0.86(0.54-1.36) | 0.519 | 0.88(0.56-1.40) | 0.601 | |
| FB^n^/F^ntotal^ | 1.21(0.86-1.69) | 0.276 | 1.22(0.79-1.88) | 0.373 | 1.01(0.66-1.56) | 0.963 | |
| FBG2^n^/(FG2^n^+FBG2^n^) | 1.33(0.95-1.87) | 0.097 | 0.86(0.52-0.40) | 0.535 | 0.64(0.39-1.06) | 0.081 | |

* Statistically significant at significant level of 0.05.

CAS:Cerebral arterial stenosis; IS: ischemic stroke.

Table S6. The false discrimination rates of 5-fold cross-validation in the three methods

| Fold | Ridge | Lasso | Step |
| --- | --- | --- | --- |
| 1 | 0.309 | 0.290 | 0.271 |
| 2 | 0.310 | 0.293 | 0.261 |
| 3 | 0.297 | 0.316 | 0.283 |
| 4 | 0.292 | 0.312 | 0.274 |
| 5 | 0.270 | 0.311 | 0.270 |
| Mean | 0.296 | 0.304 | 0.272 |

Table S7. Associations between derived glycans and inflammation markers

| Glycans | CRP | TNF-a | MMP9 |
| --- | --- | --- | --- |
| FGS/(FG+FGS) | -0.085 | -0.055 | 0.012 |
| FBGS/(FBG+FBGS) | -0.124 | 0.077 | -0.134 |
| FGS/(F+FG+FGS) | -0.125 | -0.135* | -0.026 |
| FBGS/(FB+FBG+FBGS) | -0.157 | 0.040 | -0.120 |
| FG1S1/(FG1+FG1S1) | 0.037 | 0.001 | 0.089 |
| FG2S1/(FG2+FG2S1+FG2S2) | 0.067 | 0.060 | 0.172** |
| FG2S2/(FG2+FG2S1+FG2S2) | -0.109 | 0.076 | -0.193** |
| FBG2S1/(FBG2+FBG2S1+FBG2S2) | 0.035 | 0.128 | 0.073 |
| FBG2S2/(FBG2+FBG2S1+FBG2S2) | 0.026 | 0.153* | -0.004 |
| F^total^S1/F^total^S2 | 0.056 | -0.106 | 0.203** |
| FS1/FS2 | 0.142* | -0.034 | 0.251** |
| FBS1/FBS2 | 0.008 | -0.036 | 0.026 |
| FBS^total^/FS^total^ | 0.092 | 0.150* | 0.015 |
| FBS1/FS1 | 0.062 | 0.129 | -0.002 |
| FBS2/FS2 | 0.189* | 0.102 | 0.196** |
| FBS1/(FS1+FBS1) | 0.062 | 0.129 | -0.002 |
| FBS2/(FS2+FBS2) | 0.189* | 0.102 | 0.196** |
| GP1^n^ | -0.043 | 0.066 | 0.179** |
| GP2^n^ | 0.197** | 0.062 | 0.031 |
| GP4^n^ | 0.133* | 0.188** | 0.007 |
| GP5^n^ | -0.201** | -0.144* | -0.223** |
| GP6^n^ | 0.196** | 0.095 | 0.177** |
| GP7^n^ | 0.025 | -0.066 | -0.045 |
| GP8^n^ | 0.007 | 0.037 | 0.053 |
| GP9^n^ | -0.052 | -0.023 | -0.028 |
| GP10^n^ | 0.115 | -0.026 | 0.194** |
| GP11^n^ | 0.153* | -0.016 | 0.233** |
| GP12^n^ | -0.088 | -0.188** | -0.054 |
| GP13^n^ | -0.042 | -0.056 | -0.072 |
| GP14^n^ | -0.216** | -0.201** | -0.096 |
| GP15^n^ | -0.088 | -0.162* | -0.070 |
| G0^n^ | 0.180** | 0.187** | 0.046 |
| G1^n^ | 0.038 | -0.059 | 0.116 |
| G2^n^ | -0.203** | -0.214** | -0.099 |
| F^ntotal^ | -0.022 | 0.083 | 0.052 |
| FG0^ntotal^/G0^n^ | -0.161* | 0.004 | -0.016 |
| FG1^ntotal^/G1^n^ | -0.018 | 0.059 | 0.065 |
| FG2^ntotal^/G2^n^ | -0.023 | 0.060 | 0.009 |
| F^n^ | -0.124 | -0.001 | -0.129 |
| FG0^n^/G0^n^ | -0.096 | 0.053 | -0.122 |
| FG1^n^/G1^n^ | -0.119 | 0.015 | -0.158* |
| FG2^n^/G2^n^ | -0.048 | 0.040 | 0.018 |
| FB^n^ | 0.152* | 0.015 | 0.172** |
| FBG0^n^/G0^n^ | 0.074 | -0.066 | 0.141* |
| FBG1^n^/G1^n^ | 0.121 | -0.013 | 0.169* |
| FBG2^n^/G2^n^ | 0.080 | 0.001 | -0.020 |
| FB^n^/F^n^ | 0.149* | 0.014 | 0.168* |
| F^n^/(B^n^+FB^n^) | -0.148* | -0.014 | -0.165* |
| B^n^/(F^n^+FB^n^) | -0.041 | -0.058 | -0.071 |
| FBG2^n^/FG2^n^ | 0.085 | 0.002 | -0.061 |
| FG2^n^/(BG2^n^+FBG2^n^) | -0.103 | -0.028 | 0.011 |
| BG2^n^/(FG2^n^+FBG2^n^) | 0.178** | 0.161* | 0.016 |
| FB^n^/F^ntotal^ | 0.149* | 0.014 | 0.168* |
| FBG2^n^/(FG2^n^+FBG2^n^) | 0.085 | 0.002 | -0.016 |

*Correlation is significant at the 0.05 level.

**Correlation is significant at the 0.01 level.

CRP: C-reactive protein; TNF-a: Tumor necrosis factor-alpha; MMP9: Matrix metalloproteinase-9.
